# Neural Mechanisms of Human Perceptual Learning: Electrophysiological Evidence for a Two-Stage Process

**DOI:** 10.1371/journal.pone.0019221

**Published:** 2011-04-26

**Authors:** Carlos M. Hamamé, Diego Cosmelli, Rodrigo Henriquez, Francisco Aboitiz

**Affiliations:** 1 Laboratorio de Neurociencia Cognitiva, Departamento de Psiquiatría, Escuela de Medicina, Pontificia Universidad Católica de Chile, Santiago, Chile; 2 Escuela de Psicología, Pontificia Universidad Católica de Chile, Santiago, Chile; 3 Centro Interdisciplinario de Neurociencia, Pontificia Universidad Católica de Chile, Santiago, Chile; 4 Instituto de Ciencias Biomédicas, Universidad de Chile, Santiago, Chile; 5 Lyon Neuroscience Center, INSERM U1028, CNRS UMR5292, Claude Bernard UL1, Lyon, France; Ecole Polytechnique Federale de Lausanne, Switzerland

## Abstract

**Background:**

Humans and other animals change the way they perceive the world due to experience. This process has been labeled as perceptual learning, and implies that adult nervous systems can adaptively modify the way in which they process sensory stimulation. However, the mechanisms by which the brain modifies this capacity have not been sufficiently analyzed.

**Methodology/Principal Findings:**

We studied the neural mechanisms of human perceptual learning by combining electroencephalographic (EEG) recordings of brain activity and the assessment of psychophysical performance during training in a visual search task. All participants improved their perceptual performance as reflected by an increase in sensitivity (*d'*) and a decrease in reaction time. The EEG signal was acquired throughout the entire experiment revealing amplitude increments, specific and unspecific to the trained stimulus, in event-related potential (ERP) components N2pc and P3 respectively. P3 unspecific modification can be related to context or task-based learning, while N2pc may be reflecting a more specific attentional-related boosting of target detection. Moreover, bell and U-shaped profiles of oscillatory brain activity in gamma (30–60 Hz) and alpha (8–14 Hz) frequency bands may suggest the existence of two phases for learning acquisition, which can be understood as distinctive optimization mechanisms in stimulus processing.

**Conclusions/Significance:**

We conclude that there are reorganizations in several neural processes that contribute differently to perceptual learning in a visual search task. We propose an integrative model of neural activity reorganization, whereby perceptual learning takes place as a two-stage phenomenon including perceptual, attentional and contextual processes.

## Introduction

The human brain can change its activity in order to learn novel sensory configurations thus ensuring adaptive behavior throughout life. This kind of experience-dependent modification in perception has been labeled perceptual learning [1], and its neural mechanisms have been the object of increasing interest and research. In the visual modality, one possible approach to study the neural basis of human perceptual learning is based on scalp recordings of event-related potentials (ERPs). For instance, training-dependent perceptual improvements during Vernier-like stimuli discrimination are accompanied by increases in scalp global field potential at different times after stimulus presentation [2]. Amplitude modulations have also been found in both early and late components of the visual ERP for different forms of perceptual training: for example, orientation discrimination of simple or complex stimuli yielded N1 and N2 decrements over posterior sites, together with P2 or P3 increments distributed more centro-parietally [3]. Although the absence of modifications in the P1 component is usually interpreted as a top-down modulation of stimulus processing at early stages of the neural pathway [4], [5], a recent study carefully designed to obtain C1 -the earliest component of the visual ERP- after training in a texture discrimination, found stronger amplitude for trained when compared to not-trained subjects. This suggests reorganization of neural activity at the level of early visual cortices such as V1 [6].

Despite the wealth of knowledge such studies have produced, electrophysiological studies of human perceptual learning have usually focused on the difference in neural activity between trained and untrained conditions, or with how neural responses to stimuli change after a given practice period. As informative as this approach can be regarding the final consequences of expertise on brain activity, it cannot reveal how neural activity is dynamically reorganized to improve perceptual performance. In order to do so it is necessary to track changes in brain activity that follow improvement in perceptual performance throughout the entire training process.

On the other hand, a rapidly growing approach is the spectral analysis of the EEG signal, by which oscillatory activity at different frequency bands can be described in terms of phase and amplitude. High frequency oscillatory activity in the gamma band (>40 Hz) has been found to be critical for the temporal organization of neural activity in terms of spike synchrony and cell assembly formation [7]–[9]. However, while its involvement in several cognitively relevant conditions such as visual grouping [10], [11], object representation [12], attention [13], and short term memory [14], among others [15], is well documented, the role of neural oscillations in human perceptual learning remains unknown and unexplored.

**Figure 1:**
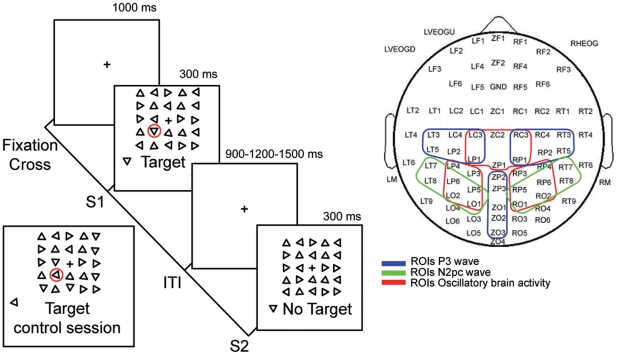
Schematic presentation of the task and electrode coverage over the scalp. The left portion of the figure depicts the visual search task’s main sequence of events. At the beginning of each block a fixation cross was presented during 1000 ms, then S1 (target present) or S2 (target absent) are presented pseudo-randomly for 300 ms. Subjects then had 1200, 1500 or 1800 ms to respond by pressing either the left or right button of a mouse if the target was present or absent from the search array respectively. This was followed by a random duration ITI that could be 900, 1200 or 1500 ms long. The bottom left panel depicts an example of the target used during the control session. The right portion of the figure shows the electrode sites spanning frontal, central, temporal, parietal and occipital areas and the identified ROIs for ERPs and neural oscillatory activity analysis.

Here we undertake the study of the neural mechanisms of human perceptual learning during the actual training process of subjects practicing a visual search task (Figure 1). Although perceptual learning has been studied mainly by training subjects to discriminate basic features of fine-grained stimuli [16], a considerable number of investigations [4], [5], [17]–[27] have used visual search tasks. The increase in ecological validity and the potential for revealing interactions between different levels of hierarchical processing suggest that such paradigm is well suited for elucidating the mechanisms of perceptual learning in the human brain. In order to follow dynamical changes in brain activity, we combined psychophysical measures and electrophysiological recordings (ERPs and oscillatory activity) during the entire processes.

Our results indicate that there is stimulus (specific) and task (unspecific) related neural activity reorganization that can be observed in amplitude changes of the N2pc and P3 ERP components, which follow a similar temporal profile as perceptual performance. On the other hand, changes in the amplitude of oscillatory activity in the gamma and alpha bands of the EEG followed a “bell” and “U” shaped pattern respectively, suggesting that the acquisition process is composed by at least two phases with distinctive optimization mechanisms for stimulus processing.

## Materials and Methods

### Ethics Statement

The Bioethical Committee of Research of the Pontificia Universidad Católica de Chile approved all experimental procedures. Research was conducted according to the principles expressed in the Declaration of Helsinki and the experiments were undertaken with the understanding and written consent of each participant. All experiments were performed at the Cognitive Neuroscience Laboratory of the Psychiatry Department of the University.

### Participants

Ten healthy human subjects (four females, six males, age range 22–35) participated voluntarily in the study. All subjects were right-handed and had normal or corrected-to-normal vision. Participants were trained in a visual search task during five sessions in five consecutive days. Each training session was also an EEG recording session and was composed of eight blocks of 150 trials. Each block lasted approximately 8 minutes. There was a five minute resting period between each block and a longer one of 15 minutes between the fourth and fifth block. Each subject was therefore exposed to 6000 trials during a training period of five consecutive days.

### Task

The visual search task used here is a modification of one used by Sigman and Gilbert [23]. All recording/training sessions took place in a dimly illuminated silent room. Stimuli were presented on a computer monitor with a 100 Hz refresh rate placed 57 cm in front of the subject's eyes. Each trial consisted in the presentation of a 300 ms search array including (S1) or not including (S2) a target, followed by a variable inter-trial interval (ITI) of 900, 1200 or 1500 ms (Figure 1A). Subjects were required to press the left or right button of a mouse in trials where they did or did not find the target respectively (two-alternative forced choice). The array subtended 4.2°×4.2° and consisted of 24 equilateral triangles and a central fixation cross. Triangles were 27′ long and had a separation of 54′ between their centers. The target triangle was defined that which presented one of the four possible orthogonal positions of their basis, and held constant across all sessions and subjects. In contrast, distractor triangles could have any of the remaining three orientations. The target was randomly presented in 50% of the trials at any of the 24 possible locations. However, since the stimuli array can be divided into two main visual eccentricities (inner and outer), 25% of the trials the target was forced to appear at one of the two possible eccentricities. Target-absent search arrays were presented in the remaining 50% of the trials. We used black triangles on a gray background in order to minimize the formation of afterimages due to the offset of a high-contrast image. Stimuli presentation and behavioral response acquisition were controlled by Presentation Package (Neurobehavioral System, Inc.). After an explanation of the task, subjects were presented with an example of both types of arrays, namely S1 and S2 and the target orientation was indicated. All subjects were instructed to respond as fast as possible, to reduce blinking and avoid eye movements during execution of the task. In order to cancel lateralized motor-related neural activity and the development of a specific neuro-motor training of one hand over the other, the response hand was alternated between successive blocks.

### Electrophysiological data

The EEG was recorded from 80 non-polarizable Ag/AgCl electrodes mounted on an elastic cap (Quick-Cap, Compumedics Neuroscan Inc.) spanning bilateral frontal, central, temporal, parietal and occipital positions as shown in Figure 1B. All sites were recorded respect to a CPZ reference electrode (here denoted as ZP2) and re-referenced off-line either to the algebraic average of the left and right mastoids (M1, M2) for ERP acquisition, or to an average of all electrodes for time-frequency analysis. Blinking, vertical and horizontal eye movements were monitored with three electrooculogram electrodes, two bellow and above the left eye and one on the external canthus of the right eye. Electrode impedance was kept bellow 10 KOhm and all the recordings were performed in a Faraday cage to reduce electromagnetic contamination. The EEG signal was acquired at a sampling rate of 1000 Hz, amplified and band-pass filtered between 1–200 Hz (NuAmps, Compumedics Neuroscan Inc.). Trials containing excessive blinking or eye movements were rejected, while other sources of signal contamination, such as myographic activity were corrected using ICA decomposition [28] and visual inspection of the data. ERPs were obtained for each condition and type of behavioral response (see bellow) by averaging over trials for each session and low-pass filtered at 30 Hz using MATLAB (Mathworks, Natick, MA, USA) toolbox EEGLAB [28]. ERP waves or components were identified on the basis of their polarity, latency and distinctive topographical properties. All ERPs were corrected respect to a −300 to −50 ms pre-stimulus baseline and the amplitude of each component or wave was calculated as the mean potential in the following time windows (ms): P1, 75-135; N1, 80-180; N2pc, 200-350; P3, 320-550. The N2pc wave, which has been consistently shown to be involved in visual search tasks, was obtained as the difference between posterior electrodes ipsi and contralateral to target position in the search array [29], [30].

Oscillatory brain activity was analyzed by means of a time-frequency (TF) representation using a Morlet wavelet transform of the signal obtained from each single trial:



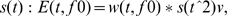
(1)where time-varying energy *E*(*t*, *f* 0) of signal *s*(*t*) in a frequency around *f* 0 band is the result of convolving its square norm with a complex wavelet *w* (*t*, *f* 0). The wavelet family was defined with a ratio of f0/σf = 7 ranging from 2 to 90 Hz. TF-representations acquired in this way were averaged across trials according to each condition and divided into four frequency bands, namely: theta (4–8 Hz), alpha (8–14 Hz), beta (20–30 Hz) and gamma (30–60 Hz). The mean power for each frequency band was normalized as amplitude *z-scores* relative to the baseline (−300 to −50 ms): in order to present brain oscillatory activity as power variations (activations or deactivations) related to stimulus presentation.



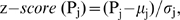
(2)with Pj, µ j and σ j, representing power, mean and standard deviation of electrode J, respectively. TF-analysis was performed with the software package for electrophysiological analysis Elan-Pack, developed at INSERM U821 (http://u821.lyon.inserm.fr/).

### Psychophysical assessment

We used signal detection theory indexes applied to psychophysics to measure the perceptual learning process. All behavioral data analysis was performed using custom designed routines in MATLAB. Responses were classified in four types: yes to S1 (hit), no to S1 (miss), yes to S2 (false alarm) and no to S2 (correct rejection). Hit and false alarm rates were calculated as: *H* = *P*(“yes”/S1) and *FA* = *P*(“yes”/S2). In order to assess perceptual improvement in visual search performance, the sensitivity index to the target was measured as:
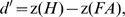
(3)where *z*(*H*) and *z*(*FA*) correspond to the inverse of the normal distribution transform of H and FA respectively. Values of H = 1 and FA = 0 were corrected to H = 0.99 and FA = 0.01 to avoid infinite values of *d'*.

With the purpose of measuring bias as the tendency to respond “yes”, a criterion index was obtained as:
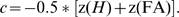
(4)


In this manner, if *FA* is equal to the miss rate, *c* = 0, if *FA* is higher or lower than the miss rate, *c* has negative or positive values respectively. Reaction times (RT) were computed as the time between search array onset and response execution.

### Statistical analysis

Because we were interested in analyzing changes related to the actual learning-acquisition process, a repeated-measures ANOVA was used to analyze the effect of training as a five-level factor (training session) on dependent variables: *d'*, RT, *c*, ERP-wave amplitude and oscillatory activity band amplitude.

Subject-level, Wilcoxon signed-rank test was performed to identify significant increases of energy in the above-mentioned frequency bands of oscillatory activity, and false discovery rate (FDR) was used to control for multiple comparisons [31]. Group level, paired samples *t-student* test was used: 1) To test the significance of the difference-wave for obtaining N2pc; 2) To decide whether to use absolute or relative values for P3 amplitude; 3) As planned pair-wise comparisons between training sessions where we expected gradual change in performance indexes; and 4) To examine if changing target orientation during the control session had an effect on psychophysical performance and brain activity.

Finally, Pearson correlation coefficient was obtained in order to test the level of dependency between psychophysical and electrophysiological data. Due to the binary nature of the task (target detected or missed) it is not possible to extract a trial-by-trial correlation leading to a block-by-block design for ERPs-psychophysics and a session-by-session design for oscillatory activity-psychophysics. All of these procedures were implemented using the statistical computational package SPSS Statistics (IBM®).

## Results

### Psychophysical data

**Figure 2:**
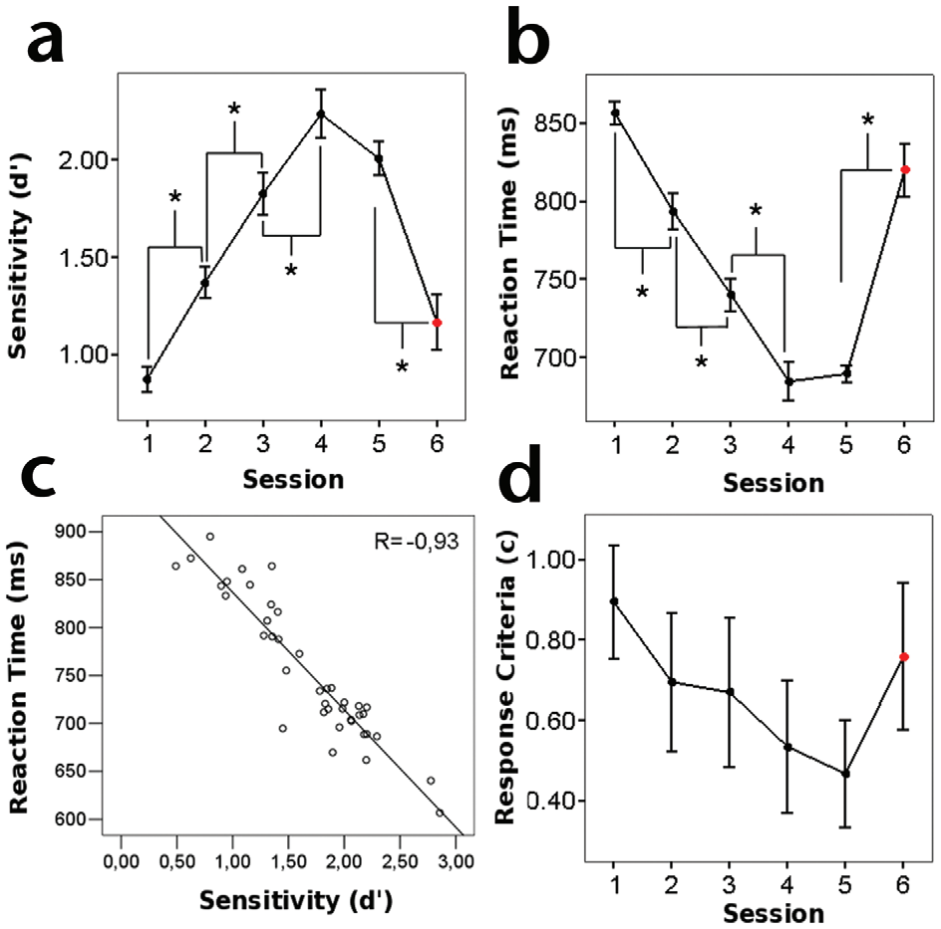
Psychophysical assessment of perceptual learning. A) Increase of mean sensitivity along training. B) Decrease of mean RT along training. C) Block-level correlation showing a dependence between sensitivity and RT. D) Response criterion along training. All significant changes between sessions are indicated by asterisks. Control sessions are indicated by a red marker.

Psychophysical results confirmed that the improvement of subject*'*s performance in the task was the result of perceptual learning. We observed a significant effect of training on sensitivity index *d'* (*F_4,45_* = 50.861, *P*<0.001; Figure 2A) with planned pair-wise comparisons, revealing a steady increase of sensitivity, particularly between first-second (*t*_9_ = −5.224, *P*<0.01), second-third (*t*_9_ = −5.544, *P*<0.001) and third-fourth (*t*_9_ = −6.586, *P*<0.001) training session. This trend did not continue between the fourth and last training session (*t*_9_ = 1.413, *P* = 0.191) suggesting that subjects reached a performance plateau. Importantly, we found that performance improvement was specific for the trained stimuli as shown by the significant decrease in sensitivity when subjects were tested using a different target orientation in a subsequent control session (*t*_9_ = 6.403, *P*<0.001). Sensitivity improvement did not occur at the expense of the speed of response: there was a significant effect of training over mean RT (*F*_4,45_ = 95.495, *P*<0.001; Figure 2B), with responses getting faster along sessions. Planned pair-wise comparisons indicated a significant steady decrease in mean RT, between first-second (*t*_9_ = 4.944, *P*<0.01), second-third (*t*_9_ = 7.864, *P*<0.001), and third-fourth (*t*_9_ = 5.722, *P*<0.001), but not between the fourth and last training session (*t*_9_ = −0.39, *P* = 0.706). As with sensitivity, mean RT changes were specific for the trained orientation and did not transfer to other targets as shown by the slower responses obtained during the control session (*t*_9_ = −7.375, *P*<0.001). Sensitivity (*d'*) and response speed (RT) measures showed a negative correlation (*R* = −0.93, *P*<0.001; Figure 2C), allowing us to use both measures jointly as an indication of perceptual learning.

Despite the fact that we did not find an effect of training on response criterion (*F*_4,45_ = 0.615, *P* = 0.655; Figure 2D), we did find a drop in *c* when comparing first and last training session (*t*_9_ = 3.832, *P*<0.005). Nevertheless, no changes were observed in false alarms (*F*_4,45_ = 0.794, *P* = 0.537; Figure S1) suggesting that the improvement in perceptual performance is most likely not related to extra-perceptual factors (e.g. decision-making or response strategy). Moreover, in contrast with sensitivity and RT, we did not find a clear trend among subjects for this index (Figure S1).

### Electrophysiological data: ERP waves

Visual ERPs to search array presentation were obtained for each subject and condition (hit, correct-rejection, miss). Because perceptual learning without transfer to novel stimuli has traditionally been interpreted as involving changes in early visual areas [1], [5], [16], we initially studied the effect of training on early components of the visual evoked response during hits. P1 and N1 waves were therefore identified over occipital electrodes peaking between 75–135 and 80–180 ms after stimulus presentation respectively. However, we did not find significant effect of training either over P1 (*F*_4,45_ = 1.821, *P* = 0.141) or N1 (*F*_4,45_ = 1.118, *P* = 0.36) amplitude.

**Figure 3:**
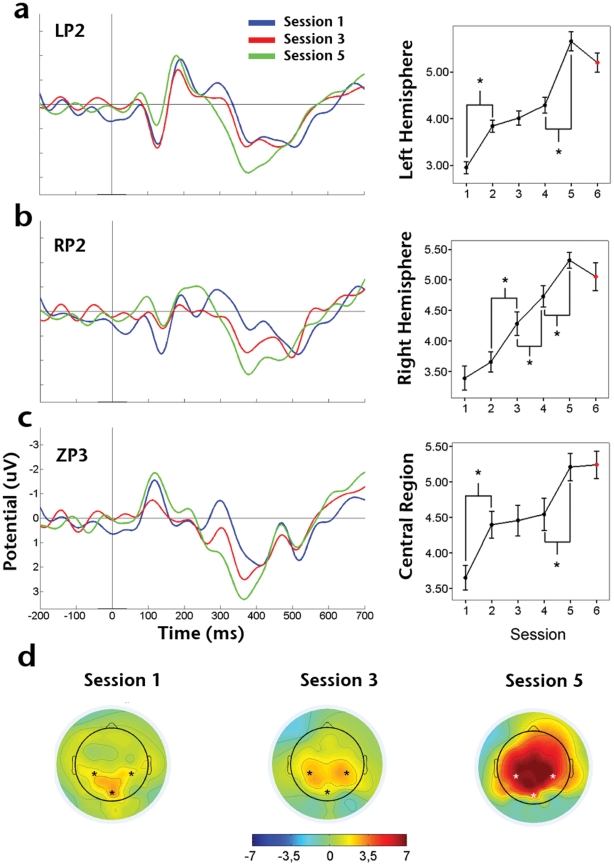
Effect of training over mean P3 amplitude. Grand average ERPs obtained from session 1, 3 and 5 (sessions 2 and 4 are omitted for clarity) and from electrodes: A) LP2, left parietal; B) RP2, right parietal; C) ZP3, central mid-line. D) Average topographic distribution of scalp potential peak in the P3 time window for the corresponding sessions.

Late ERP waves P3 and N2pc were processed in a slightly different fashion due to the high sensitivity they show to target presence:

A P3b-like (from now on P3 for simplicity) component was identified by a rather distributed scalp topography, which appeared more clearly over central and parietal sites, showing no significant differences between target and no-target trials (supplementary Figure S2). We analyzed the absolute value of P3 amplitude throughout the perceptual learning process, and found a significant effect of training as measured over left (*F*_4,45_ = 123.42, *P*<0.001; Figure 3A), right (*F*_4,45_ = 39.631, *P*<0.001; Figure 3B) and central (*F*_4,45_ = 105.841, *P*<0.001; Figure 3C) posterior electrodes. Planned pair-wise comparisons revealed that this main effect could be described as a tendency of P3 amplitude to increase over the course of learning. More specifically, in left electrodes, the amplitude increment was significant between first-second (*t*_9_ = −6.74, *P*<0.001) and fourth-fifth (*t*_9_ = −13.185, *P*<0.001) training sessions. In right-hemisphere electrodes, significant differences were found between sessions second-third (*t*_9_ = −8.068, *P*<0.001), third-fourth (*t*_9_ = −4.893, *P*<0.001) and fourth-fifth (*t*_9_ = −4.519, *P*<0.01). Finally, in the case of central electrodes, significant differences were found between sessions first-second (*t*_9_ = −0.2638, *P*<0.05) and fourth-fifth (*t*_9_ = −10.392, *P*<0.001). In contrast to behavioral indexes, however, the increment in P3 amplitude was not specific for target orientation as revealed by the absence of significant differences between the last and control sessions, where target orientation had been modified (left: *t_9_* = −0.321, *P* = 0.756; right: *t_9_* = 0.369, *P* = 0.721; and central: *t_9_* = 1.985, *P* = 0.078).

The N2pc component has been widely involved in visual search tasks such as the one used in the present study, and has been targeted as a neurophysiological marker for selective processing of stimuli that are embedded in sets of distractors [29], [30]. However, despite the fact that visual search tasks have been frequently used to explore visual perceptual learning [4], [5], [17]–[27], there have not been, to our knowledge, any reports analyzing the relationship between N2pc and this process. In order to isolate the N2pc component, we obtained the difference wave between ipsi and contralateral-to-target potentials (see Methods). We found a significant difference waveform in the 200 to 350 ms post-stimulus time-window, for all sessions in both left and right posterior electrodes (lower *P* value<0.05; supplementary Figure S3).

**Figure 4:**
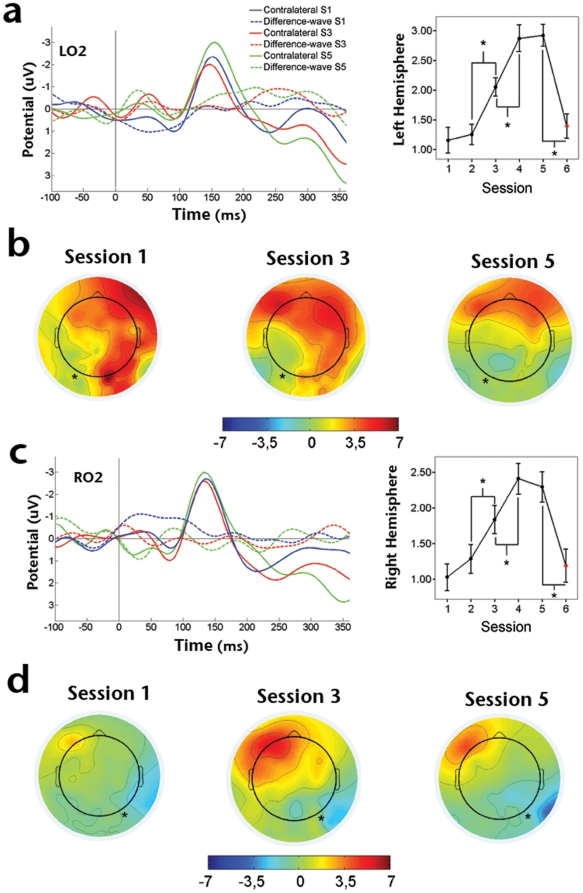
Effect of training over mean N2pc amplitude. A) Grand average ERPs contralateral-to-target and difference -waves (contra minus ipsilateral) obtained from sessions 1, 3 and 5 and from electrode: A) LO2 left occipital; B) Average topographic distribution of scalp potential peakin the N2pc time window for the corresponding sessions and for targets appearing at the right side of the array; C) RO2, right occipital; D) Same as in B but for targets appearing at the left side of the array.

There was a clear effect of training on N2pc amplitude spanning left (*F*_4,45_ = 107.629, *P*<0.001; Figure 4A) and right hemisphere (*F*_4,45_ = 68.98, *P*<0.001; Figure 4C) electrodes. Planned pair-wise comparisons showed a progressive amplitude increment of this component. In the case of the left hemisphere, significant differences were found between first-second (*t*_9_ = −2.72, *P*<0.05), second-third (*t*_9_ = −4.427, *P*<0.005), third-fourth (*t*_9_ = −5.995, *P*<0.001), but not fourth-fifth (*t*_9_ = 0.92, *P* = 0.381) training sessions, while for right sites the significant differences were found between second-third (*t*_9_ = −8.319, *P*<0.001), third-fourth (*t*_9_ = −6.085, *P*<0.001), but not for first-second (*t*_9_ = −0.667, *P* = 0.522), nor forth-fifth (*t*_9_ = −0.452, *P* = 0.662) sessions. Importantly, N2pc amplitude increase was found to be highly specific for the trained target orientation as evidenced by the significant drop in amplitude during the control session (*t*_9_ = 16.036, *P*<0.001; *t*_9_ = 9.45, *P*<0.001, for left and right sites respectively).

### Electrophysiological data: Time-frequency analysis

TF-representations were obtained trial-by-trial for each condition, normalized by subject and then averaged across epochs to construct theta (4–6 Hz), alpha (8–14 Hz), beta (20–30 Hz) and gamma (30–60 Hz) profiles of band-limited power variations. From a subject-level statistics point of view, there were significant changes in power relative to baseline activity in the alpha and gamma, but not in the theta or beta frequency bands for all subjects and training sessions in all the regions of interest (ROIs, See Methods). Accordingly, in the following we limit our analysis to the former frequency ranges only.

**Figure 5:**
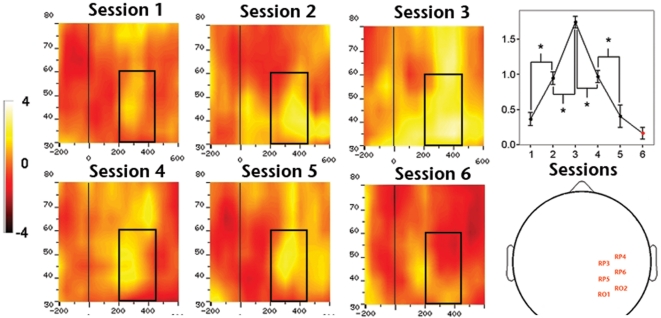
Effect of training on mean GBA amplitude over ROI-2 or right hemisphere sites. The left side of the figure depicts TF charts obtained for each of the five training sessions. The right side of the figure depicts, from top to bottom, the TF chart obtained for the control session, head plot showing the approximate position of electrodes identified for ROI-2 and quantification of the amplitude profile along training (1-5) and during control (6) session.

**Figure 6:**
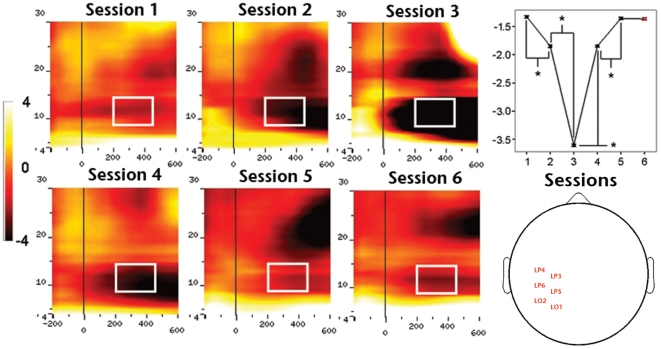
Effect of training on mean ABA amplitude over ROI-1 or left hemisphere sites. The left side of the figure depicts TF charts obtained for each of the five training sessions. The right side of the figure depicts, from top to bottom, the TF chart obtained for the control session, head plot showing the approximate position of electrodes identified for ROI-1 and quantification of the amplitude profile along training (1-5) and during control (6) session.

At the group-level, we found a significant effect of training on amplitude of gamma band activity (GBA, Figure 5) over posterior left (*F*_4,45_ = 515.851, *P*<0.01), central (*F*_4,45_ = 248.533, *P*<0.01) and right (*F*_4,45_ = 612.081, *P*<0.01) electrodes. Planned pair-wise comparisons showed a significant amplitude increment between first-second and second-third training sessions, and a significant drop between third-fourth and fourth-fifth sessions over all analyzed regions (all *P*<0.01), revealing a complex bell-shaped amplitude profile of GBA throughout the learning process. We did not find significant GBA amplitude differences between the last and control sessions for any of the ROIs (lowest *P* value >0,08). The second main group-level result concerning oscillatory brain activity was the significant effect of training on the amplitude of alpha band activity (ABA; Figure 6) over posterior left (*F*_4,45_ = 40174.531, *P*<0.01), central (*F*_4,45_ = 60133.456, *P*<0.01) and right (*F*_4,45_ = 38796.946, *P*<0.01) electrodes. Planned pair-wise comparisons showed a significant amplitude decrement between first-second (*P*<0.005) and second-third (*P*<0.01) training sessions, and a significant increment between third-fourth (*P*<0.01) and fourth-fifth (*P*<0.001) sessions in all the analyzed regions (*P*<0.05). Compared to the case of GBA, ABA also showed a complex amplitude modulation pattern along training, although this time as a mirror U-shaped profile. We did not observe significant amplitude differences between the last and control sessions for none of the assessed ROIs for ABA either. Interestingly, we found a strong inverse correlation (e.g. ROI-2: *R* = −0.993, *P*<0.001) between GBA and ABA amplitudes (supplementary Figure S4).

### Relationship between neural activity reorganizations and perceptual learning

In order to analyze the level of dependency between perceptual learning and observed changes in brain activity, we calculated the Pearson correlation coefficient between both sensitivity and mean RT, versus neural activity measures (ERP wave amplitude and oscillatory activity).

**Figure 7:**
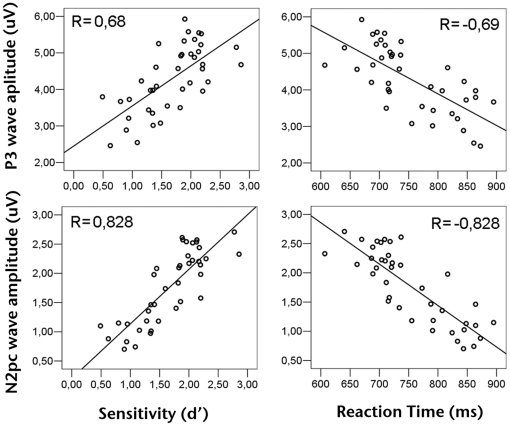
Relationship between ERP waves amplitude and perceptual learning. Top left panel shows the block-level correlation between P3 amplitude and sensitivity. Top right panel shows the block-level correlation between P3 amplitude and RT. Bottom left panel shows the block-level correlation between N2pc amplitude and sensitivity. Bottom right panel shows the block-level correlation between N2pc amplitude and RT.

We found a significant positive correlation between P3 amplitude and sensitivity (Figure 7A) over left (*R* = 0.69, *P*<0.001), central (*R* = 0.68, *P*<0.001) and right (*R* = 0.5, *P*<0.001) electrodes. Furthermore, there was a significant negative correlation between P3 amplitude and mean RT (Figure 7B) over left (*R* = −0.75, *P*<0.001), central (*R* = −0.69, *P*<0.001) and right (*R* = −0.56, *P*<0.001) electrodes. In the case of N2pc amplitude, there was an even stronger positive correlation with sensitivity (Figure 7C) over left (*R* = 0.807, *P*<0.001) and right (*R* = 0.828, *P*<0.001) electrodes, and also a clear negative correlation with mean RT (Figure 7D) over left (*R* = −0.822, *P*<0.001) and right (*R* = −0.828, *P*<0.001) electrodes.

Summarizing, as sensitivity increased and mean RT decreased throughout the training process we observed a clear trend of increase in P3 and N2pc amplitudes. On the other hand, and as was expected from the amplitude profiles of oscillatory neural activity throughout training, we found no significant correlations between either GBA or ABA amplitude and sensitivity or mean RT.

## Discussion

Taken together, the present results show that training in a visual search task produces specific and measurable changes in neural activity associated with the process of perceptual learning. It seems, however, that the relation between perceptual learning and changes in brain activity is not straightforward. Indeed, while amplitude changes found in late components of the visual ERP point to a reorganization of neural networks involved in target detection and other context-related processes, modifications in alpha and gamma band oscillatory activity indicate the presence of two phases in learning acquisition. In other words, the observed relationship between psychophysical and electrophysiological results is inconsistent with a single neurophysiological process that could account for perceptual learning, but rather suggests a more complex scenario of dynamically interacting neural reorganizations. In the following we discuss the main results and propose a neurophysiological model to account for our findings.

Our behavioral results confirm the occurrence of perceptual learning in the visual search task as evidenced by an improvement in sensitivity and a reduction in RT throughout training (see Figure 2A,B). This improvement was found to be significant from first to fourth, but not between fourth and fifth sessions, suggesting that perceptual performance reached an asymptotic level towards the end of training. When analyzing the subject's response criterion, on the other hand, we did not find a significant effect of training as revealed by the ANOVA design, suggesting that the training process did not modify the tendency of the subjects to indicate the presence or absence of the target. However, an apparent drop was observable in the overall pre-post comparison. A possible explanation for this would be that, albeit minimally, subjects increased FAs as they progressed through the task due to increased confidence. However, as shown in the supplementary Figure S1b, FAs maintained a constant level, thus ruling out that subjects became more prone to signal the presence of the target. Because *c* is calculated as the negative average of the *z-scores* of HR and FA (see Eq. 4), this could explain the progressive but non-significant drop of *c* seen in Figure 2a, given that HR systematically increases with training (see supplementary Figure S1a), while FAs stay unchanged. Finally, the behavioral improvements we observed were specific for the trained target orientation as can be seen from the drop in sensitivity and increase in RT when testing with a non-trained orientation in the control session. This is in agreement with results from the original version of this visual search paradigm, whereby practice-related enhancements in performance were shown to be specific for the trained object and orientation [23].

Our ERP results show training-dependent changes in the amplitude of ERP waves P3 and N2pc, but not for P1/N1. Such lack of modulation in early components may seem in contradiction with previous studies suggesting a link between low degree of learning generalization of learned stimuli and changes in early stages of the visual path. However, such studies have used fine-grained stimuli, presented always at the same location and with the relevant dimension consisting of a single basic feature, such as line orientation or position. In contrast, we trained subjects to find a simple shape in an array of distractors, a task that is unlikely to produce modifications in early visual areas because of the small receptive fields of its neurons [32]. Additionally, because targets could appear at different locations within the array it is possible that the resulting potentials cancelled out in the averages. Finally, and in agreement with fMRI studies of perceptual learning [33], triangles orientation may have been perceptually easy to determine, therefore leading to little or no reorganization in early stages of visual processing yet inducing more pronounced changes in later ones [5].

Although practice had a consistent effect on P3 amplitude, it turned out to be non-specific for the trained target, as revealed by the results of the control session (see Figure 3). This change cannot be attributed to habituation [34] or attention [35], since, on one hand, repetitive stimuli presentation leads to a decrease rather than an increase of P3 amplitude, while, on the other, an increase in RTs as the one observed during the control session (Figure 2b) would be incompatible with a general target-independent attentional facilitation. Previous studies have found training-dependent increments in the amplitude of the P3 wave, but they did not evaluate the level of specificity by testing subjects' performance with untrained stimuli [3]. If the amplitude increment of P3 is correlated with performance, but not specifically related to the trained stimulus, the underlying changes in neural activity could be responsible for some other relevant aspect of task execution. We propose that modulation in P3 could reflect an unspecific task- or process-based learning [36] that is boosting performance through diffuse reinforcement signals [37]. This kind of task-based learning interpretation for P3 amplitude modulation is consistent with early (and most widely accepted) theories for the functional role of the P3 wave, according to which the rather heterogeneous cognitive conditions that affect P3 amplitude can be grouped under the concept of context update [38]. This would imply a process-based learning that is enhancing performance through the optimization of activity related to context update (i.e. updating of information regarding stimulus and general environmental conditions), but not to the specific identity of the target. Optimization of unspecific task-based processes is also consistent with previous behavioral evidence [39], [40] and recent models of guided search comprising not only selective, but also non-selective pathways for target identification depending on its context [41].

The present results show for the first time that sustained practice in a visual search task leads to amplitude increments in the N2pc wave that are strongly correlated with psychophysical performance and highly specific for trained stimulus orientation. N2pc is involved in selective processing of targets embedded in complex visual arrays [29], [30]. Accordingly, training-dependent amplitude increments of this component could represent a neurophysiological correlate of an improvement in this capability. The specificity level exhibited by this activity modification, however, argues against a purely attentional-related effect and suggests that performance improvements should be at least partially dependent upon visual cortices capable of responding to specific stimuli features (e.g. orientation, shape). Changes in N2pc amplitude could therefore be the result of the dynamical interaction between sensory cortices and upstream attentional networks, accounting both for specificity and for classical N2pc results in visual search tasks. Indeed, Ahissar and Hochstein [4], [5] have proposed a mechanism for such interaction in which attention constrains the stimuli attributes upon which learning takes place. Furthermore, to account for performance improvements outside the focus of attention [42], the original model was extended by proposing that perceptual learning occurs thanks to the coincidence of diffusive reinforcement signals related to task execution (also in agreement with our P3 results) and signals induced by target presentation [43]. In this context, attention would work as a gate mechanism, selecting which aspects of the task will be learned with a lower or higher degree of generalization [37].

Our ERP results fit well in the framework provided by Ahissar and Hochstein's model, but suggest several relevant lines of development. We propose that unspecific, context-related effects of practice can be seen through a P3 amplitude increment, while both unspecific and specific attention-related effects are associated with changes in the N2pc profile. Indeed, Hopf and collaborators have shown that the N2pc component has its origins in both parietal and occipito-temporal cortices [44]. This would be compatible with an interaction between fronto-parietal attentional networks [45] and sensory cortices resulting in attentional re-weighting that encompasses an increase in the amount of attention paid to perceptual dimensions and important features, and/or a withdrawal of attention from irrelevant aspects of the stimulus [46].

In addition to behavioral and ERP results, we found novel evidence of the effects of sustained training on posterior oscillatory brain activity for both gamma and alpha frequency bands. Interestingly, their temporal profiles were not monotonic but showed complex amplitude patterns better described as bell- and U-shaped respectively. Previous investigations regarding human perceptual learning and GBA have found a decrease in the case of priming [47], and an increase in the case of associative [48] and rapid perceptual learning [49]. However, these studies compared GBA either pre- vs post-training or trained vs non-trained, without assessing the entire course of learning acquisition. Moreover, such studies did not focus on perceptual learning as the result of extensive practice in a sensory task.

We have also ruled out the possibility that there could be an oculomotor explanation for our GBA results (see supplementary Text S1 and supplementary Figure S5), as it has been recently suggested to be the case of most scalp EEG studies [50]. Indeed, we believe that GBA as the one measured here is a relevant neurophysiological phenomena related to local and/or large-scale neuronal synchrony and cell assembly conformation [7], [8]. If brain oscillations are related to neural assemblies formation by affecting the probability of temporal coincidence of unitary spikes relative to the phase of each oscillation cycle [9], then it is possible to link training-dependent GBA amplitude modifications to changes in the conformation of functional cell ensembles. This has long been considered as one of the most probable neural mechanisms for learning [51]. In the specific case of our paradigm, the progressive increment of GBA from the first to the third training session could reflect an increase in the strength and/or the number of synaptic connections, promoting a better signal-to-noise ratio and therefore improving perceptual performance. The following progressive decrease in GBA, on the other hand, is compatible with a second form of boosting target detection by means of an overall synaptic downscalling leading to the establishment of a sparse code. Here, only neurons with the most strong and/or selective response would remain as part of the responsive cell assembly, thus increasing coding efficiency. In this context, it is important to note that, although this is the first report on the role of oscillatory neural activity in perceptual learning, the finding of an increase-followed-by-a-decrease pattern of amplitude in neural activity has been previously found in fMRI studies that showed similar training dependent modifications in V1 [52] and regions of the ventral visual pathway [53]. Given the strong coupling between GBA and BOLD signal [54], [55], we believe the convergence between our results regarding brain oscillatory activity and these previous fMRI studies is quite significant.

Compared to GBA, ABA followed a mirror profile of amplitude changes across training, dropping initially but then increasing after the third session. Despite being the most prominent EEG rhythm, the functional role of alpha is still debated among the alternatives of cortical idling, inhibition or active top-down control hypotheses [56], [57]. For example, regarding attentional orienting, a decrease in ABA has been found over contralateral-to-shift parietal cortex [58], but also an increase over parietal areas ipsilateral-to-shift when using a behaviorally relevant no-shift condition as control [59]. A clearer picture, however, may be drawn in the case of visual discrimination, where it has been shown that higher levels of pre-stimulus ABA correlates with lower performance in the task [60]. Given the complexity of this rhythm, it is certainly possible that ABA plays different roles depending on the task at hand [56].

In our study, subjects were trained to perform a visual search that involved both attention and discrimination and we observed an inverse correlation in the amplitude of ABA and GBA throughout the process (supplementary Figure S3). Taking into account the previous considerations regarding the putative roles of GBA and ABA, this leads us to propose that the present results may be revealing complementary properties of high neural excitability-GBA and low neural excitability-ABA during learning acquisition. This would mean that while GBA is reflecting the degree in which cells are spiking in a more packed way (thus forming neural ensembles), ABA may be indicative of a similar, but sparser organization of neural activity in time.

**Figure 8:**
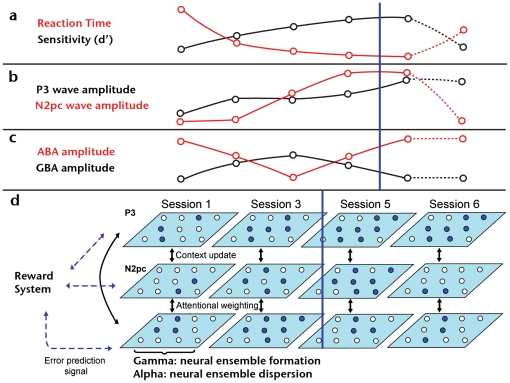
Neurophysiological model for visual perceptual learning. A) Schematic representation of the psychophysical results. B) Schematic representation of ERPs results. C) Schematic representation of oscillatory neural activity results. D) Simplified cartoon representing neural activity reorganizations proposed in the model as the neurophysiological basis of perceptual learning. Each panel represents putative neural networks placed on different brain regions. Empty circles represent inactive neurons or neural populations, while filled circles represent active neurons or neural populations. Arrows represent activity modulations among the different neural networks. For A, B and C, the blue lines mark the moment around which perceptual performance becomes saturated.

There is probably more than one mechanism [52], [53] by which different brain networks [27], [33] reorganize their activity in order to produce the characteristic psychophysical improvements of perceptual learning. The present results suggest that neural reorganization due to perceptual learning is a multi-layered phenomenon involving different mechanisms at different stages of acquisition. Figure 8 depicts a simplified model that aims at integrating previous accounts of perceptual learning and the present results. In our model, both specific and unspecific learning take place along the entire course of training. The unspecific aspects are reflected in P3 amplitude, and comprise the development of a cognitive configuration relative to the task and its context, enhancing performance by facilitating non-selective context updating processes [36], [37], [41]. Specific processes are reflected in the N2pc amplitude, favoring detection and identification of the trained target by means of a differential weighting and re-weighting mechanism [46]. This would take place at a mid-level stage of perceptual processing [52] where an interaction between attentional neural networks and sensory cortices can facilitate the selective treatment of the target among distractors. Finally, oscillatory brain activity over posterior sites would reflect underlying changes at an early-local network stage [61]. This would enhance performance by augmenting the number and/or strength of connections in a given cell ensemble during a first phase and by selecting only the most strong and selective neurons during the second one. Importantly, and in agreement with our results, previous fMRI reports [52], [53] also show that the second phase starts around the same time that perceptual improvement becomes asymptotic. Although the proposed orchestration of neurophysiological processes is still probably an incomplete scheme of experience-dependent changes in brain activity, we expect that highlighting the dynamical modifications that take place during training will suggest and open possible directions for future research.

## Supporting Information

Figure S1 **Single subject psychophysical profiles along training.** A) Hit rate, B) False alarm rate, C) Sensitivity or *d'* D) Response criterion or *c.*
(TIF)

Figure S2 **Comparison of P3 amplitude between hit and correct-rejection conditions.** Grand-averaged ERPs obtained from a left parietal site showing no significant differences in P3 amplitude for hit and omission trials in sessions: A) One, B) Two, C) Three, D) Four, E) Five and F) Control.
(TIF)

Figure S3 **N2pc calculation.** Grand-averaged ERPs obtained from an occipito-lateral site, specifically showing potentials obtained from trials with ipsi (blue) and contralateral (red) apparition of the target in sessions: A) One, B) Two, C) Three, D) Four, E) Five and F) Six or “control”. The N2pc component amplitude is defined as the difference between posterior contra and ipsilateral evoked potential (black).
(TIF)

Figure S4 **Relationship between GBA and ABA amplitude along training.** There was a strong dependency between GBA and ABA amplitude as revealed by a significant session-wise negative correlation in A) ROI-1 (left sites), B) ROI-2 (right sites) and C) ROI-3 (central sites).
(TIF)

Figure S5 **Comparison between GBA amplitude obtained in conditions hit and correct rejection.** TF charts constructed from the difference between hot- and correct-rejection trials for training sessions and control. Original TF charts were constructed from ROI-3 or central electrode sites.
(TIF)

Text S1 (DOC)
