# Supplementary figures and images for: Neural Mechanisms of Human Perceptual Learning: Electrophysiological Evidence for a Two-Stage Process
Source: PLoS One. 2011 Apr 26;6(4):e19221. doi: 10.1371/journal.pone.0019221 (PMC3082555; doi:10.1371/journal.pone.0019221)

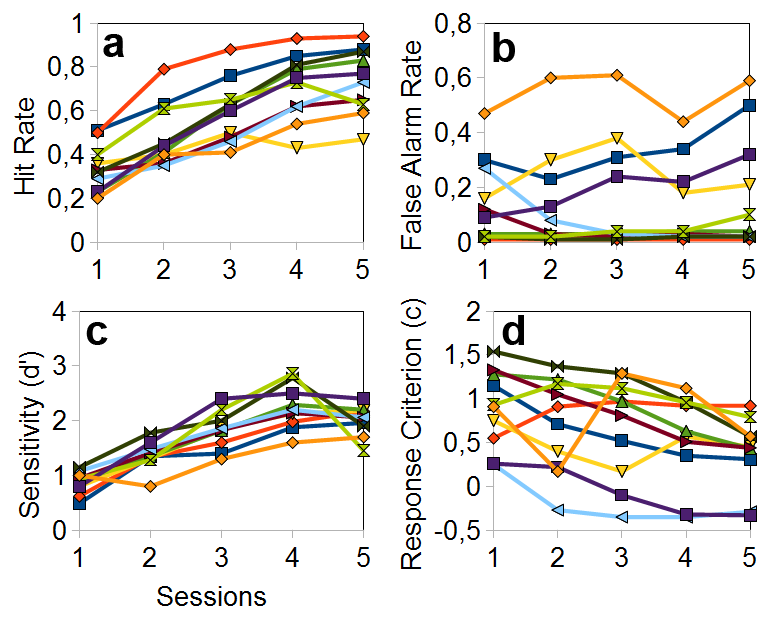

Supplement: Figure S1 — Single subject psychophysical profiles along training. A) Hit rate, B) False alarm rate, C) Sensitivity or d' D) Response criterion or c. (TIF) [file pone.0019221.s001.tif]

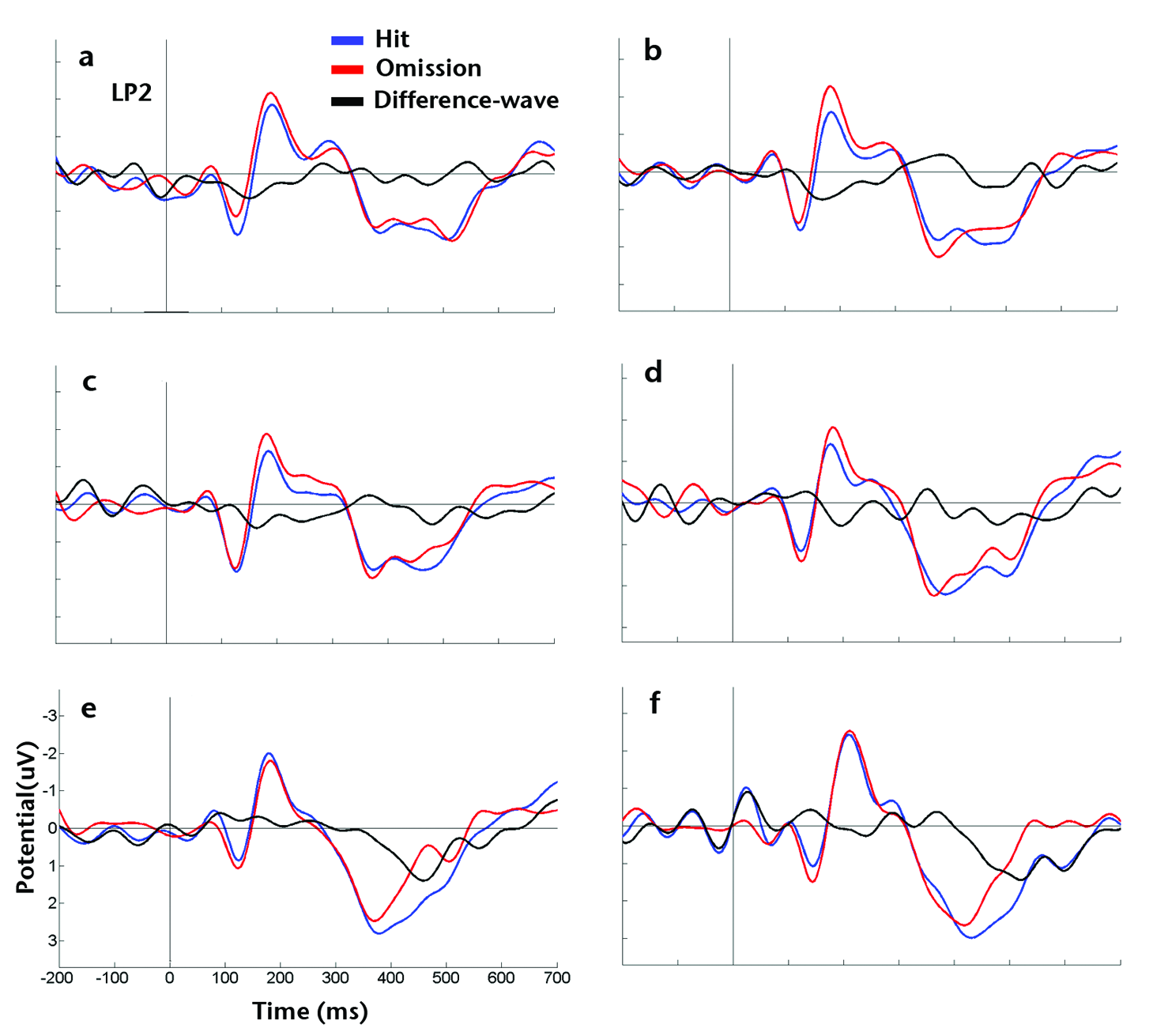

Supplement: Figure S2 — Comparison of P3 amplitude between hit and correct-rejection conditions. Grand-averaged ERPs obtained from a left parietal site showing no significant differences in P3 amplitude for hit and omission trials in sessions: A) One, B) Two, C) Three, D) Four, E) Five and F) Control. (TIF) [file pone.0019221.s002.tif]

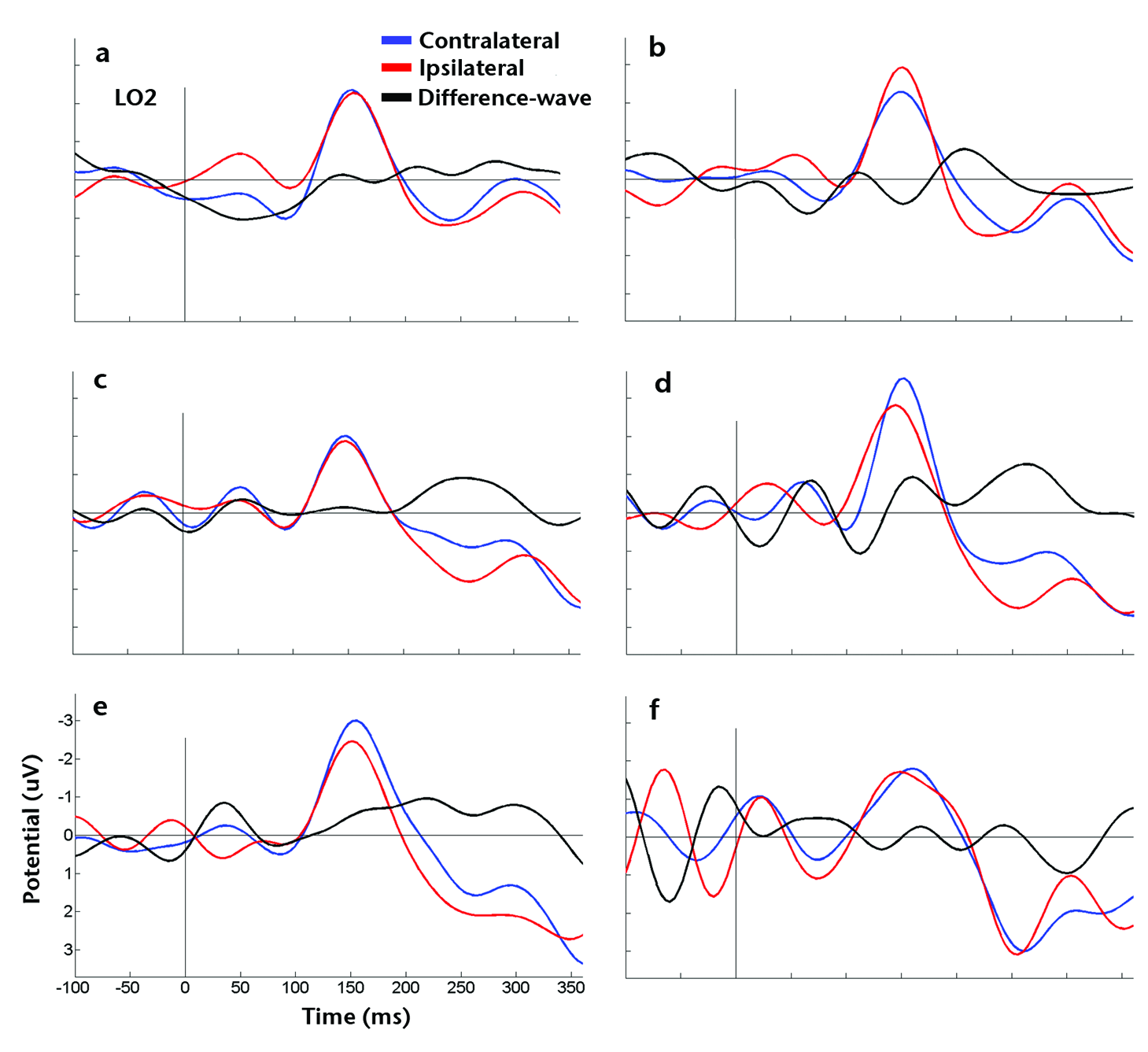

Supplement: Figure S3 — N2pc calculation. Grand-averaged ERPs obtained from an occipito-lateral site, specifically showing potentials obtained from trials with ipsi (blue) and contralateral (red) apparition of the target in sessions: A) One, B) Two, C) Three, D) Four, E) Five and F) Six or “control”. The N2pc component amplitude is defined as the difference between posterior contra and ipsilateral evoked potential (black). (TIF) [file pone.0019221.s003.tif]

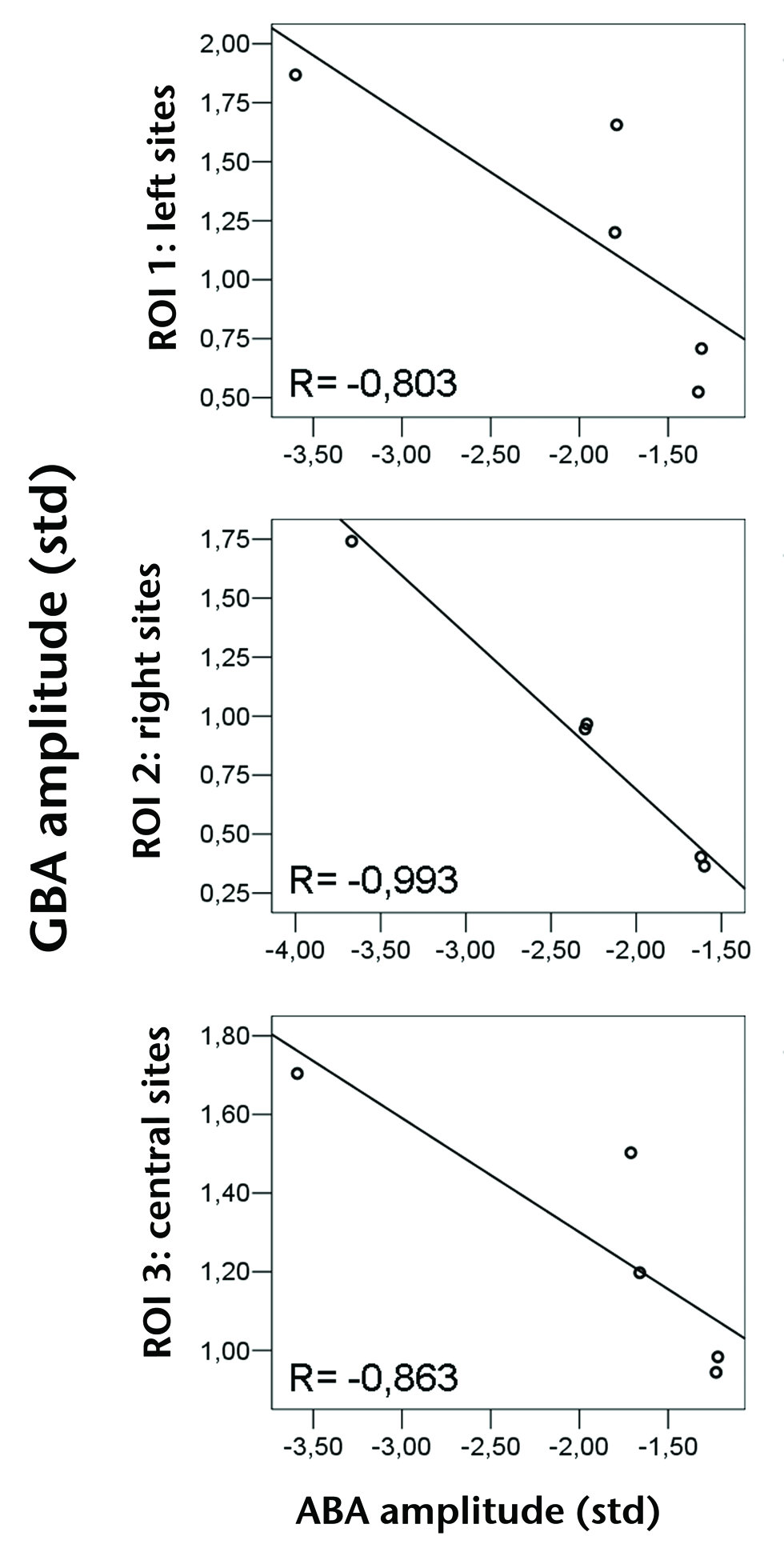

Supplement: Figure S4 — Relationship between GBA and ABA amplitude along training. There was a strong dependency between GBA and ABA amplitude as revealed by a significant session-wise negative correlation in A) ROI-1 (left sites), B) ROI-2 (right sites) and C) ROI-3 (central sites). (TIF) [file pone.0019221.s004.tif]

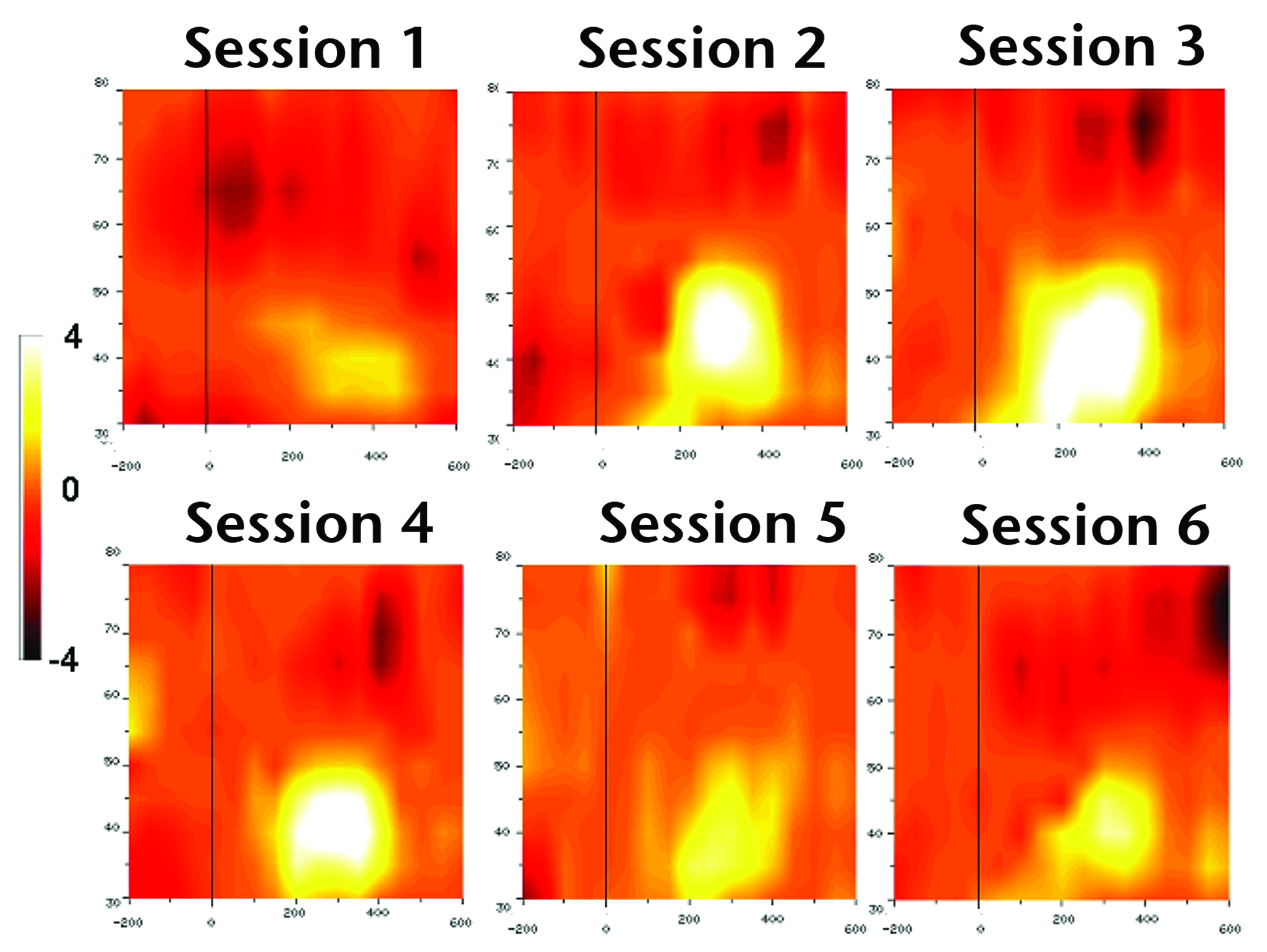

Supplement: Figure S5 — Comparison between GBA amplitude obtained in conditions hit and correct rejection. TF charts constructed from the difference between hot- and correct-rejection trials for training sessions and control. Original TF charts were constructed from ROI-3 or central electrode sites. (TIF) [file pone.0019221.s005.tif]
