# Supplementary material for: Neural Mechanisms of Human Perceptual Learning: Electrophysiological Evidence for a Two-Stage Process
Source: PLoS One. 2011 Apr 26;6(4):e19221. doi: 10.1371/journal.pone.0019221 (PMC3082555; doi:10.1371/journal.pone.0019221)
Supplement: Text S1 — (DOC) [file pone.0019221.s006.doc]

**Neural mechanisms of human perceptual learning: electrophysiological evidence for a two-stage process**

Carlos M. Hamame ^1,3,4,5^, Diego Cosmelli ^1,2,3^, Rodrigo Henriquez ^1,3^, Francsico Aboitiz ^1,3^

1. Departamento de Psiquiatría, Escuela de Medicina; and Centro Interdisciplinario de Neurociencia, Pontificia Universidad Católica de Chile, Santiago, Chile.

2. Escuela de Psicología, Pontificia Universidad Católica de Chile, Santiago, Chile.

3. Centro Interdisciplinario de Neurociencia, Pontificia Universidad Católica de Chile, Santiago, Chile.

4. Instituto de Ciencias Biomédicas, Universidad de Chile, Santiago, Chile.

5. Lyon Neuroscience Center, INSERM U1028, CNRS UMR5292, Claude Bernard UL1, Lyon, France.

Correspondence should be addressed to CMH (carlos.hamame@inserm.fr)

**Supporting Text S1**

**Supporting References**

**SUPPORTING TEXT S1**

**GBA training-dependent modifications are not reflecting microsaccades**

It has been recently suggested that, while MEG or intracortically acquired GBA is a neural response, transient increases of GBA power measured through scalp-EEG can be explained by miniature eye movements or microsaccades [1,2]. Concerned by this claim, the present study took two approaches in order to demonstrate the neural origin of the dynamics for GBA amplitude along training. The first one was to use an average-reference, this was made considering the fact that microsaccade-contamination of the EEG signal it is the result of polar references such as nose tip, frontal pole or mastoids [3-5]. This is clearly not enough to preclude such contamination, so the second approach took advantage of an unexpected control condition which was based on the comparison of hit- vs correct rejection-trials. There is no reason to think that there will be more eye movements when the target is present than when the target is absent in the search array. In fact, it is highly probable that subjects would perform more eye movements during trials with no target than during trials where the target was present, this is because the no-target condition will pose a higher demand for a serial than a parallel search strategy [6,7].

Figure S4 shows that even though it was more probable to have microsaccadic contamination when target was absent than when target was present, GBA was significantly higher for hit-trials than for correct-rejection trials, demonstrating that the here recorded EEG-GBA has a neural and not an artefactual origin.

**REFERENCES**

1. Yuval-Greenberg S, Tomer O, Keren AS, Nelken I, Deouell LY (2008) Transient induced gamma-band response in EEG as a manifestation of miniature saccades. Neuron 58: 429-441.

2. Yuval-Greenberg S, Deouell LY (2009) The broadband-transient induced gamma-band response in scalp EEG reflects the execution of saccades. Brain Topogr 22: 3-6.

3. Trujillo LT, Peterson MA, Kaszniak AW, Allen JJB (2005) EEG phase synchrony differences across visual perception conditions may depend on analysis and recording methods. Clin Neurophysiol 116: 172-189.

4. Melloni L, Schwiedrzic CM, Rodriguez E, Singer W (2009) (Micro)Saccades, corollary activity and cortical oscillations. Trends Cogn Sci 13: 239-245.

5. Melloni L, Schwiedrzic CM, Wibral M, Rodriguez E, Singer W (2009) Response to: Yuval-Greenberg et al., ‘‘Transient induced gamma-band response in EEG as a manifestation of miniature saccades.’’ Neuron 58: 429–441. Neuron 62: 8-10.

6. Treisman A, Gelade G (1980) A feature integration theory of attention. Cog Psychol 12: 97-136.

7. Sigman M, Gilbert CD (2000) Learning to find a shape. Nat Neurosci 3: 264-269.
